# Genomic profiles and transcriptomic microenvironments in 2 patients with synchronous lung adenocarcinoma and lung squamous cell carcinoma: a case report

**DOI:** 10.1186/s12920-020-0663-8

**Published:** 2020-01-31

**Authors:** Licheng Wu, Poming Kang, Shaolin Tao, Zhikun Zhao, Longyun Chen, Yajie Xiao, Qunyou Tan

**Affiliations:** 1 Department of Thoracic Surgery, Daping Hospital, Third Military Medical University, Chongqing, China; 2 YuceBio Technology Co., Ltd., Shenzhen, China

**Keywords:** Lung squamous cell carcinoma, Lung adenocarcinoma, Multifocal lung cancer, Genetic profiles, Transcriptomic microenvironments

## Abstract

**Background:**

Multifocal lung cancers (MLCs) are common in patients newly diagnosed with lung cancer, and histological results of most synchronous MLCs are similar. Few cases with different histology findings have been reported, and no genomic or transcriptomic profiling of this kind of cases were done before. Here, we analyzed genomic and transcriptomic profiles of all lung tumors from 2 patients with synchronous adenocarcinoma and squamous cell carcinoma in the same lung lobe.

**Case presentation:**

Two patients were diagnosed as synchronous adenocarcinoma and squamous cell carcinoma and underwent surgical resection. All 4 tumors showed distinct genomic profiles, therefore were independent primary tumors. Several cancer-associated pathways, such as RTK-RAS pathway and Notch pathway, exhibited different mutated genes in different tumors from the same patient. Several known cancer genes with different mutations, including *TP53* and *KEAP1*, were also detected. Mutation signature analysis demonstrated that the tumor initiation might be related to the transcription coupled nucleotide excision repair process. Two tumors for these 2 patients had loss of heterogeneity (LOH) in HLA genes, showing tumor escaping mechanism. Furthermore, tumor microenvironments showed different patterns in 2 tumors from the same patient. The tumor with more neoantigens and no HLA LOH showed more infiltrating CD8+ T cells and more clonal TCRs, indicating a more active microenvironment.

**Conclusions:**

The lung squamous cell carcinoma and lung adenocarcinoma form the same patient are from independent origins. The genetic profiles and transcriptomic microenvironments are quite different for these 2 tumors. With the same genetic background, the 2 tumors in one patient exhibited different tumor escape mechanisms and immune responses, including HLA LOH and T cell infiltrating and expansion.

## Background

Lung cancer is the leading cause of cancer incidence and mortality in China and all over the world [1]. Among all lung cancer patients, about 0.7–15% of them are diagnosed as multifocal lung cancers (MLCs) [2–6] for having two or more distinct primary tumors in different sites of single or both lungs. According to the occurring time, the MLCs can be divided into synchronous or metachronous lung cancers. Histological results of most of the synchronous MLCs are similar, whereas some of the metachronous MLCs are different due to the cancer transforming. Besides, patients with MLCs might also have intrapulmonary metastases with similar morphology. Therefore, accurate clinical diagnosis is very difficult and very crucial for making treatment decisions.

The synchronous MLCs represent complex evolutionary process. Some synchronous MLCs occur independently, but some others are originated form a single site and then spread to other sites [3, 7, 8]. The inter-tumor heterogeneity and clonal architecture in the same patients of synchronous MLCs have been well documented [7, 8]. The tumors in independent MLCs have distinct genomic profiles and might be driven by different events [8]. Besides, the tumors with the same origin undergo a complex evolutionary process, and tumors might compete with each other [7]. However, only a few cases have been reported as synchronous MLCs with different histology findings [4]. The molecular characteristics of synchronous MLCs still remain unclear.

In this study, we analyzed 4 tumors from 2 patients with synchronous lung adenocarcinoma and lung squamous cell carcinoma to evaluate genetic profiles and transcriptomic microenvironments. Our results demonstrated that lung squamous cell carcinoma and lung adenocarcinoma form the same patient were from independent origins, showing quite different genetic profiles and transcriptomic microenvironments.

## Case presentation

### Patients description

**Fig. 1:**
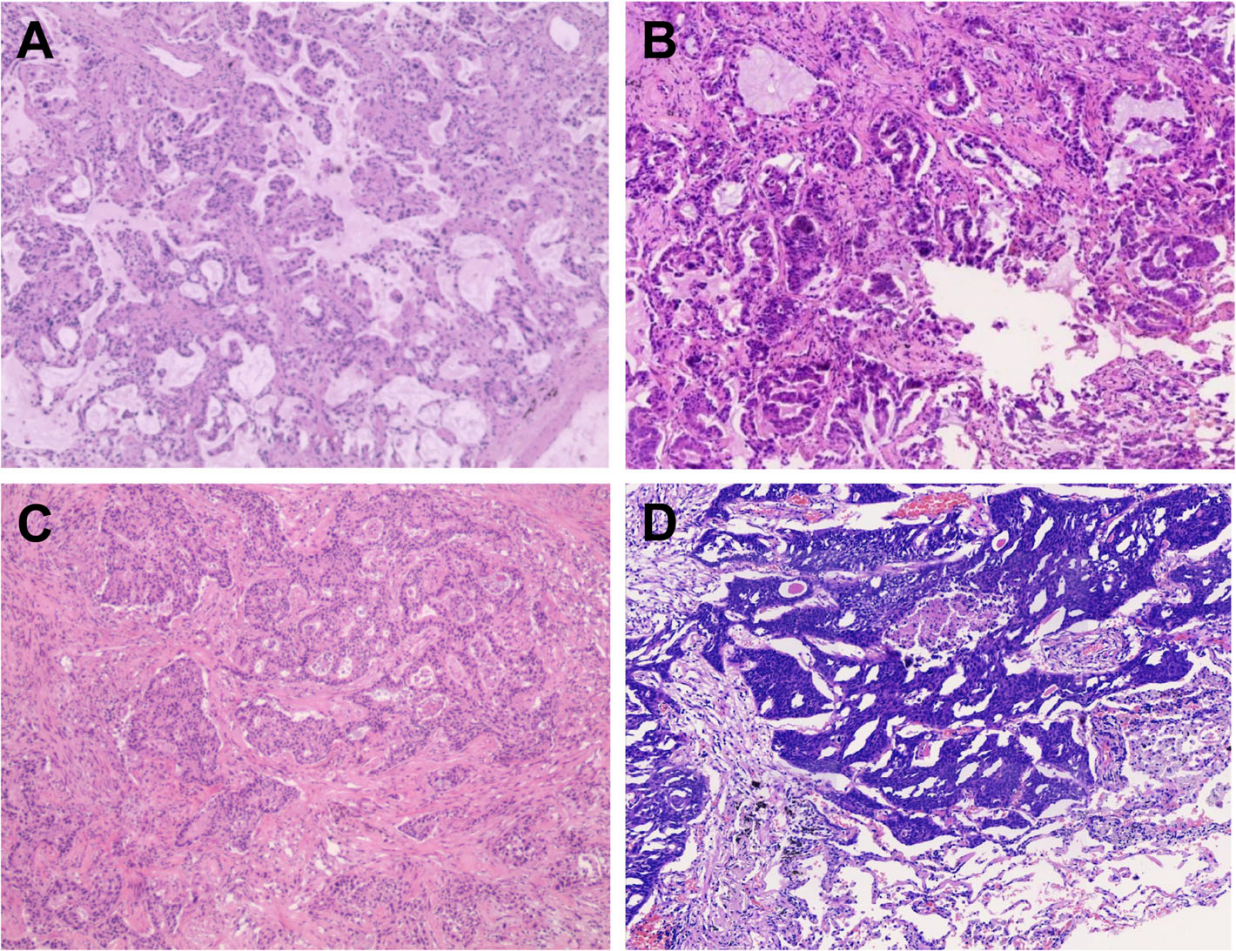
Pathological features of both patients. H & E staining (X100) of **a** P1A, **b** P1S, **c** P2A, (D) P2S

Two patients were diagnosed with pathologically confirmed synchronous MLCs and were free of extrathoracic metastases. The two tumors from the same patients were all on the same lung lobe. The patients underwent surgical resection, and the tumor sizes ranged from 1.3 to 1.6 cm according to pathology reports (Table 1). The excised tumors were confirmed as adenocarcinoma and squamous cell carcinoma morphologically and immunohistochemically (Fig. 1).

**Table 1:** Patient characteristics

Patient ID	Age	Gender	Smoking status	Tumor ID	Size (cm)	Location	Histology	TNM stage
P1	72	Male	Smoker	P1A	1.6	RUL	ADC	pT1cN0M0
				P1S	1.6	RML	SQCC	pT1cN0M0
P2	77	Male	Former smoker	P2A	1.3	LUL	ADC	pT1bN0M0
				P2S	1.6	LUL	SQCC	pT1bN0M0

*RUL* right upper lobe, *RML* right middle lobe, *LUL* left upper lobe, *ADC* adenocarcinoma, *SQCC* squamous cell carcinoma

**Fig. 2:**
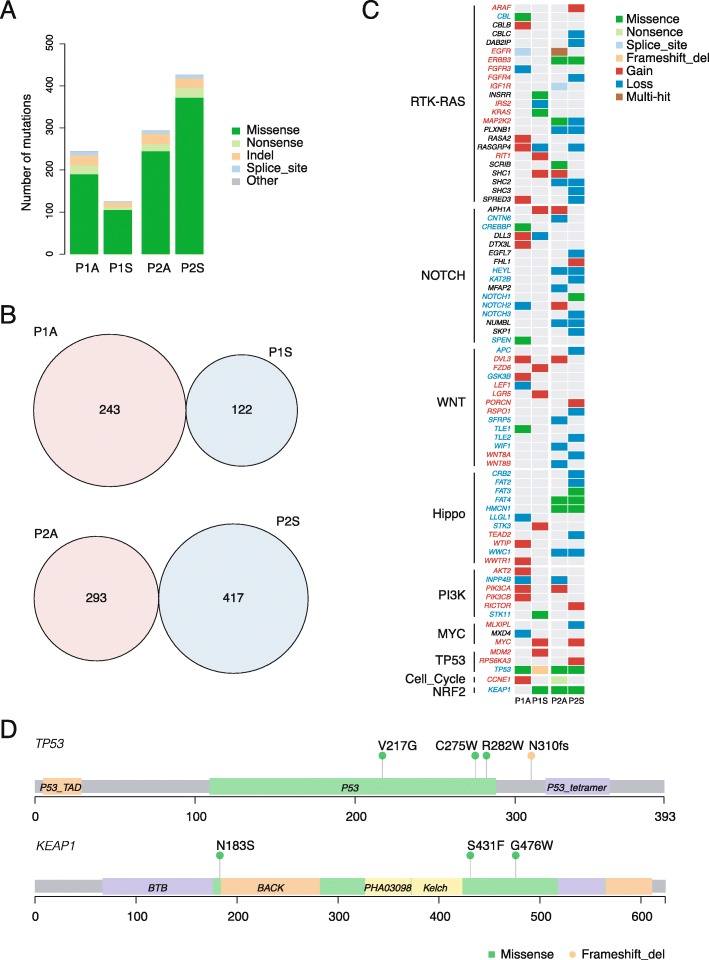
Mutations and CNVs. **a** Numbers of non-silent somatic mutations detected in the tumor samples. **b** Overlap of non-silent mutations of adenocarcinoma and squamous cell carcinoma samples from the same patient. **c** Mutations and CNVs in genes of known oncogenic pathways. The colors of gene names denote gene types: red, oncogene; blue, tumor suppressor gene; black, unknown. **d** Point mutations and indels of *TP53* and *KEAP1*

To investigate the developmental mechanism of the synchronous lung adenocarcinoma and lung squamous cell carcinoma, we performed WES on tumor samples and adjacent normal samples at a mean of 230x depth. Approximately 99% bases of the target region were covered with a depth of 10x or more. A total of 1092 non-silent somatic mutations were detected, including 912 missense mutations, 67 nonsense mutations, 83 insertions or deletions and 30 other types of mutations (Additional file 3: Table S1). The mutational burdens varied between patients and between cancer types (range from 122 to 417 per sample) (Fig. 2a).

No shared mutations were detected between ADC and SQCC from the same patient, indicating that the ADC and SQCC were all primary tumors (Fig. 2b). We also found several pathways harbored genes with SNVs or CNVs, including RTK-RAS pathway, Notch pathway, WNT pathway, Hippo pathway, PI3K pathway, MYC pathway, TP53 pathway, cell cycle pathway and NRF2 pathway [9] (Fig. 2c). Alterations in these pathways could result in the tumor proliferation, differentiation and inhibition of apoptosis. A total of 81% genes in these pathways had CNVs, indicating chromosomal instability might occur in these early stage tumors.

A total of 96 mutated genes (range from 16 to 37 per sample) of these pathways were detected. Of these genes, 73% genes existed SNVs or CNVs in only one sample. Tumors of P1 showed amplifications of oncogenes in ADC and SQCC, while tumors of P2 occurred deletions of tumor suppressor genes in ADC and SQCC (*p* < 0.05) (Fig. 2c). We detected several recurrent mutated genes, including *TP53*, *KEAP1*, *ERBB3*, *FAT4* and *HMCN1*. However, all mutated sites were different among the 4 tumors, indicating a convergent mutational process (Fig. 2d).

**Fig. 3:**
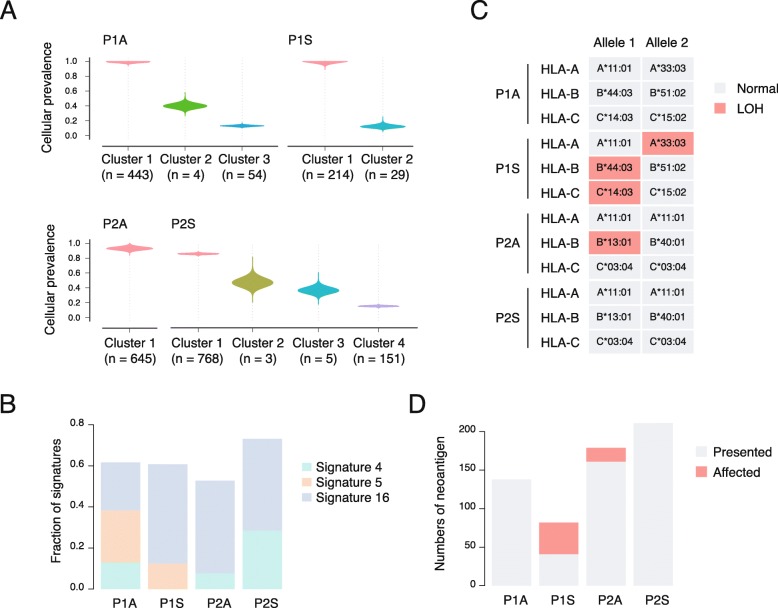
Tumor clonality and tumorigenesis. **a** Mutation clones detected in 4 tumors. **b** Mutation signatures. **c** Loss of heterogeneity (LOH) of HLA alleles. **d** Influence of HLA LOH on predicted neoantigens

To better understand the initiation of the tumors, we analyzed the clonality of the 4 tumors using silent and non-silent mutations (Fig. 3a). The intra-tumor heterogeneity was at a low level, as expected. Approximate 89% of mutations were clonal mutations (range from 82 to 100%). The clonal architectures of the two tumors of P1 were similar (Fig. 3a). But the P2A and P2S showed distinct clonal structures. P2A were homogeneous with only one clone, while P2S exhibited 3 subclones, indicating a more complex evolution (Fig. 3a). We further examined the mutations of cancer genes in Fig. 1c. Most of the mutations (93%) were clonal mutations, suggesting the potential role of the pathways during tumor initiation.

The mutation spectra of SNVs were consistent across all the samples, with a preponderance of C > A, followed by C > T (Additional file 1: Figure S1). The Signature 4 was found in 3/4 tumors (P1A, P2A and P2S). This signature has been reported to be associated with tobacco exposure [10], consistent with the smoking history of these 2 patients (Fig. 3b). We found a high fraction of Signature 16 across all 4 samples. The proposed aetiology of Signature 16 remains unknown. This signature exhibits a strong transcriptional strand bias for T > C mutations at ApTpN context and is observed in hepatocellular carcinomas [10]. It’s reported that Signature 16 might be introduced by transcription coupled nucleotide excision repair (NER) as the potential outcome of bulky DNA helix-distorting adducts on adenine [10]. Furthermore, Signature 5 was detected in P1A and P2A (Fig. 3b). This signature is similar with Signature 16, showing a strong transcriptional strand bias. These indicated Signature 16 and its similar signatures might play an important role during the tumorigenesis of the synchronous lung adenocarcinoma and lung squamous cell carcinoma of these two patients.

Intriguingly, we found loss of heterogeneity (LOH) of human leukocyte antigen (HLA) in P1S and P2A (Fig. 3c). It’s known that HLA LOH is an important approach for tumor cells to escape immune surveillance and attacking [11]. Although the adenocarcinoma and the squamous cell carcinoma might occur through similar mechanism, the mechanism of immune escaping might be different. Previous study showed the HLA LOH tended to occur during the late stage of tumor evolution and preferred to affect the presentation of neoantigens derived from sub-clonal mutations [11]. Therefore, we predicted neoantigens and analyzed the clonality of neoantigens. HLA LOH impaired the presentation process of 50% neoantigens in P1S, and the fraction in P2A was 10% (Fig. 3d). All the affected neoantigens were derived from clonal SNVs or indels. This might because the tumor resection was done at an early stage. Thus, the tumors undertook a low immune pressure, and did not have enough time to acquire sub-clonal mutations.

**Fig. 4:**
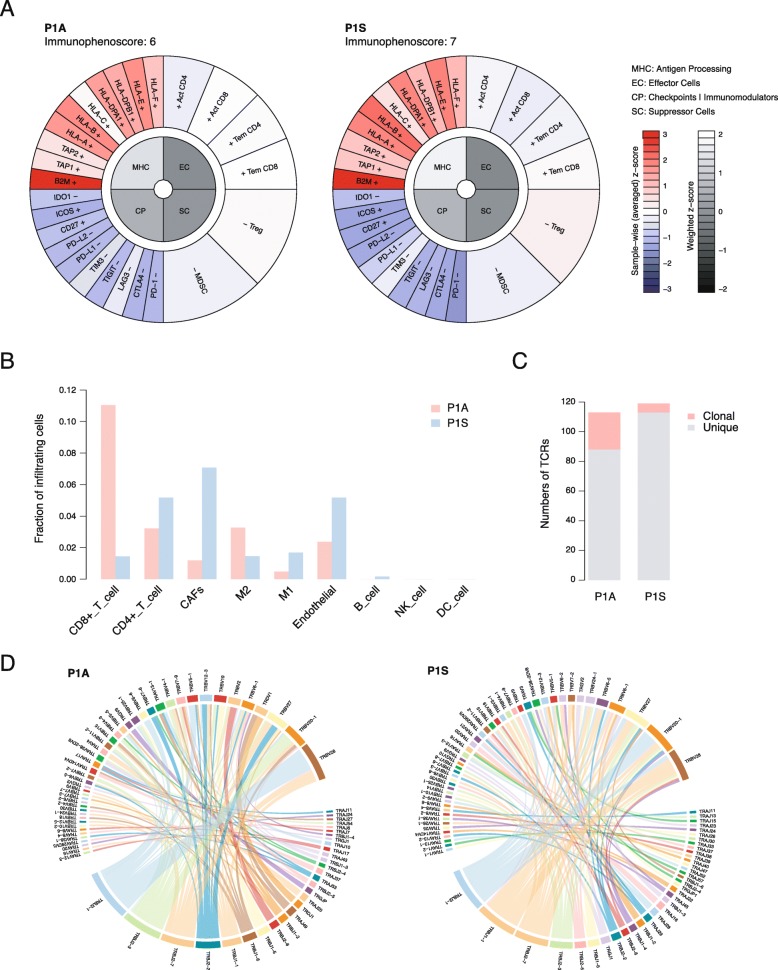
Tumor microenvironments. **a** Immunophenoscore of P1A and P1S. **b** Fractions of infiltrating cells. CAFs, cancer-associated fibroblasts. M1, macrophage M1. M2, macrophage M2. NK cell, natural killer cells. DC cell, dendritic cell. **c** Numbers of T cell receptors (TCRs). **d** Distribution of TCR V gene and J gene usages

Tumor microenvironment (TME) evolved by tumor surrounding cells, including immune cells, fibroblasts, endothelial cells and other kinds of cells. We performed RNA-sequencing to investigate on the tumor microenvironments between P1A and P1S (Additional file 3: Table S2). We first characterized the immune signatures using the Immunophenoscore (IPS) [12] (Fig. 4a). Using 20 single immune factors and 6 immune cell types, the IPS divided the tumor microenvironment into four aspects: antigen processing (MHC), effector cells (EC), checkpoints I immunomodulators (CP) and suppressor cells (SC). Compared to P1S, P1A had higher scores of CP and EC, indicating a more active TME.

We then inferred the proportions of infiltrating cells by the deconvolution of the RNA expression (Fig. 4b). The fraction of CD8^+^ T cells in P1A was 6-fold higher than that in P1S, while the fraction of CD4^+^ T cells in P1S was slightly higher, consisting with the IPS results (Fig. 4b). The different distributions of T cells between two tissues were validated by immunohistology (Additional file 2: Figure S2). Meanwhile, P1S exhibited more non-immune infiltrating cells, including cancer-associated fibroblasts (CAFs) and endothelial cells, which were reported to contributed to the tumor progression (Fig. 4b). These discrepancies might directly influence tumor progression between P1S and P1A. Additionally, the numbers of TCR clones were comparative in P1A and P1S (113 vs 119) (Fig. 4c). However, the proportion of clonal TCRs in P1A was significantly higher than that in P1S (22% vs 3%, Fisher’s exact test, *p* < 0.01), indicating more T cell expansion events in P1A (Fig. 4c). Among all the TCRs, only one TCR (CASRVSVGAKEQYF) was detected in two samples. And the usages of V genes and J genes were quite different between the two samples (Fig. 4d). These might due to the entirely different sets of neoantigens.

## Discussion and conclusions

Although comprehensive analysis of the genetic and transcriptomic characteristics of lung adenocarcinoma and lung squamous cell carcinoma have been done, no such analysis has been performed on patients diagnosed with synchronous adenocarcinoma and squamous cell carcinoma.

In this study, the adenocarcinoma and squamous cell carcinoma from the same patient were all primary tumors without shared somatic mutations. All four samples exhibited massive copy number gain and loss, indicating the chromosomal instability. Although the mutated genes were different, the pathways affected by the SNVs and CNVs were similar. All 4 samples exhibited highest fractions of mutated genes in RTK-RAS pathway. The RTK-RAS pathway has highest median frequency of alterations across all tumor types, as well as in lung cancer [9, 13–19]. Activation of this pathway can result in unlimited proliferation, cell survival and abnormal translation [9, 13–19]. We also found high fractions of mutated genes in WNT and Notch pathways, which were all well-known oncogenic pathways [9]. Interestingly, copy number gains in oncogenes were more frequently in P1, while losses in tumor suppressor genes were more frequently in P2. Besides, several tumor suppressor genes, including *TP53*, *KEAP1*, *FAT4* and *HMCN1* were recurrently mutated in tumors with distinct mutated sites. These indicated that tumors might alter the functions of the same oncogenic pathways through different approaches, such as mutation in the same genes, or copy number alterations in the same pathways. Except the similar altered pathways, the mutation signatures of the four samples were also similar. Despite the smoking-associated signatures, there were high fractions of Signature 5 and Signature 16 related to transcription coupled NER. Although Signature 16 were common in hepatocellular carcinomas, it might relate to the tumor initiation of this rare type of lung cancer.

In addition to the alterations in cancer genes and oncogenic pathways, the changes related to immune response were also very crucial. For the tumor cells, we detected LOH of three HLA alleles in P1S and one allele in P2A. For P1S, the antigen presentation was affected in about 50% of the predicted neoantigens. This might reduce the neoantigens presented to the tumor cell surface, leading to the escape from the immune surveillance at a very early stage. Furthermore, using RNA-sequencing, we deciphered the tumor microenvironment of P1A and P1S. Compared to the P1S, P1A showed a more active immune response, with a higher immune signature score of effector cells, more CD8^+^ T cells infiltrating, and more fraction of clonal TCRs. This discrepancy might due to the HLA LOH in P1S. As a result, less tumor cells might be recognized by CD8^+^ T cells, leading to a relative cold microenvironment. Besides, there were more infiltrating CAFs in P1S. It has been reported that CAFs could contribute to the suppression of anti-tumor T-cell responses by driving the death and dysfunction of tumor specific T cells [20]. This was consistent with the cold microenvironment in P1S.

With the two patients, we identified the difference of tumor initiation of adenocarcinoma and squamous cell carcinoma, as well as the interaction between tumor and TME. To better understand the tumorigenesis and immune response, we will expand the cohort in further analysis.

Our study characterized detailed genomic and transcriptomic features of synchronous lung adenocarcinoma and lung squamous cell carcinoma at first time. The genomic profiles demonstrated that these two lesions from the same patients were all primary tumors. Although the histological findings were different, the altered pathways were similar. However, HLA LOH was only detected in one of two tumors in both two patients, indicating distinct immune escaping mechanisms. Besides, the TMEs of P1 showed various proportions of infiltrating stroma and immune cells and various degrees of T cell expansion, revealing different immune responses under the distinct tumor contents.

## Supplementary information

**Additional file 1: Figure S1.** Mutational spectra of four tumor samples. The mutational type proportion for each substitution in a trinucleotide context is shown (total 96 contexts).

**Additional file 2: Figure S2.** Immunohistology of CD8^+^ and CD4^+^ T cells in P1 (X400). (A) CD8^+^ T cells of P1A. (B) CD8^+^ T cells of P1S. (C) CD4^+^ T cells of P1A. (D) CD4^+^ T cells of P1S.

**Additional file 3: Table S1.** Non-synonymous mutations of P1 and P2. **Table S2.** Gene expressions (FPKM) of P1.

## Data Availability

The datasets in this study are included in this article and its supplementary information files. The sequencing data or other datasets are available from the corresponding author on reasonable request.

## Abbreviations

ADC: Adenocarcinoma
CAF: Cancer-associated fibroblast
CNV: Copy number variation
CP: Checkpoints or immunomodulators
EC: Effector cells
IPS: Immunophenoscore
LOH: Loss of heterogeneity
LUL: Left upper lobe
MHC: Antigen processing
MLC: Multifocal lung cancer
RML: Right middle lobe
RUL: Right upper lobe
SC: Suppressor cells
SNV: Single-nucleotide variation
SQCC: Squamous cell carcinoma
TCR: T cell receptor
TME: Tumor microenvironment
WES: Whole exome sequencing
