# Supplementary material for: Genomic profiles and transcriptomic microenvironments in 2 patients with synchronous lung adenocarcinoma and lung squamous cell carcinoma: a case report
Source: BMC Med Genomics. 2020 Jan 31;13:15. doi: 10.1186/s12920-020-0663-8 (PMC6995067; doi:10.1186/s12920-020-0663-8)
Supplement: Supplementary file 1 — Additional file 1: Figure S1. Mutational spectra of four tumor samples. The mutational type proportion for each substitution in a trinucleotide context is shown (total 96 contexts). [file 12920_2020_663_MOESM1_ESM.pdf]

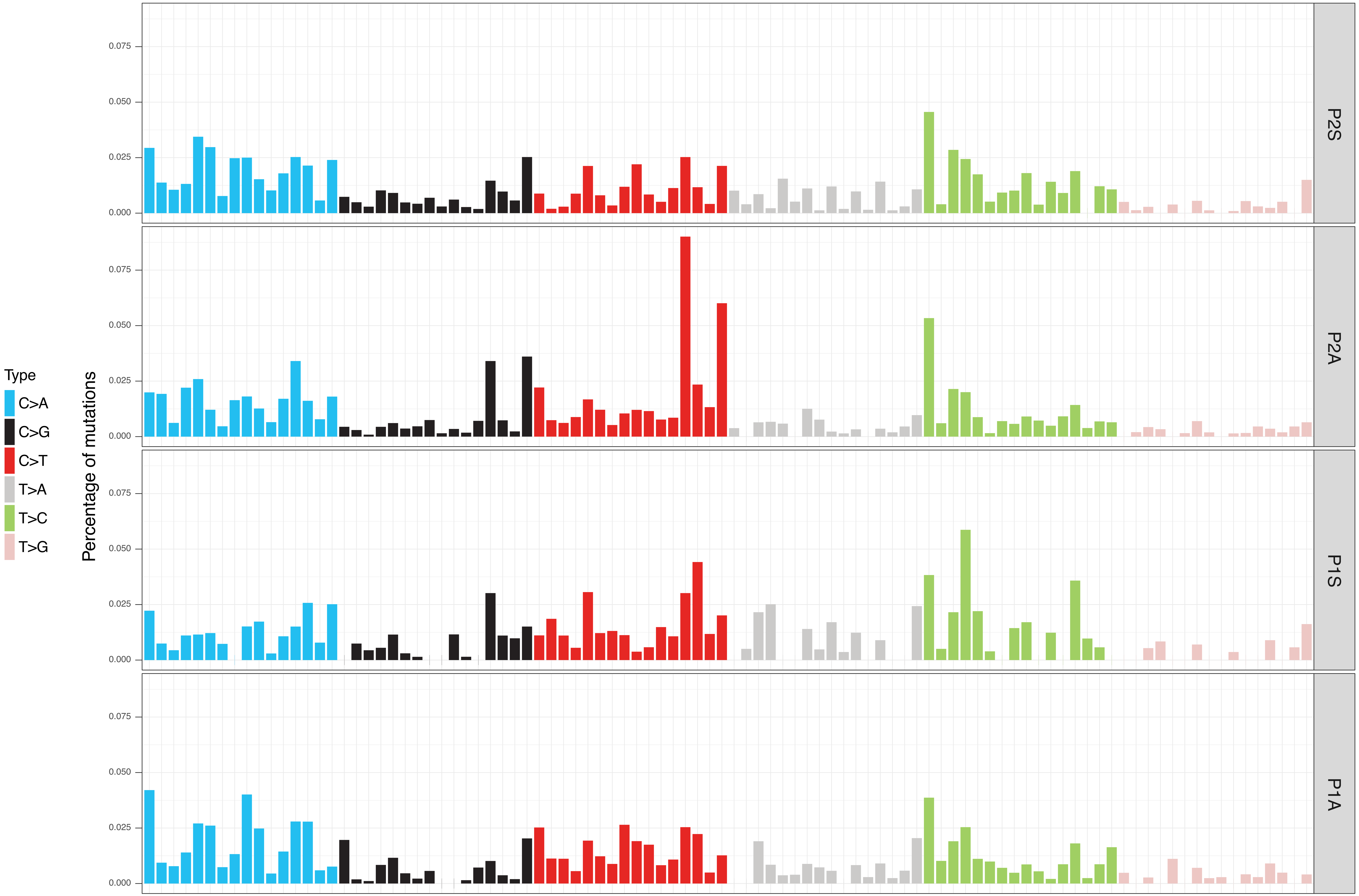
Type
C>A
C>G
C>T
T>A
T>C
T>G
Percentage of mutations
0.075
0.050
0.025
0.000
P2S
P2A
P1S
P1A
