# Supplementary figures and images for: Genomic profiles and transcriptomic microenvironments in 2 patients with synchronous lung adenocarcinoma and lung squamous cell carcinoma: a case report
Source: BMC Med Genomics. 2020 Jan 31;13:15. doi: 10.1186/s12920-020-0663-8 (PMC6995067; doi:10.1186/s12920-020-0663-8)

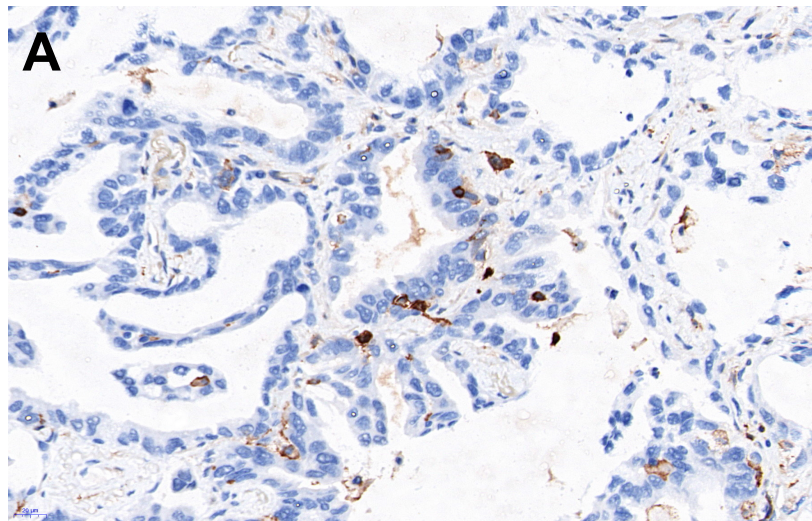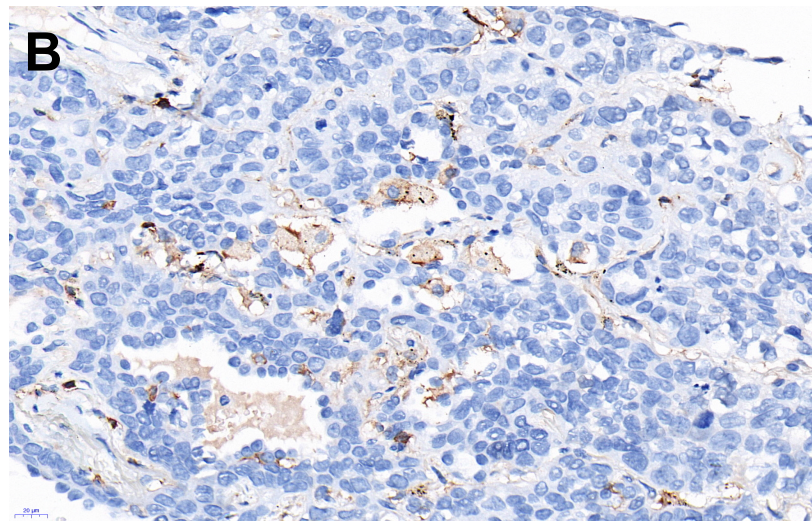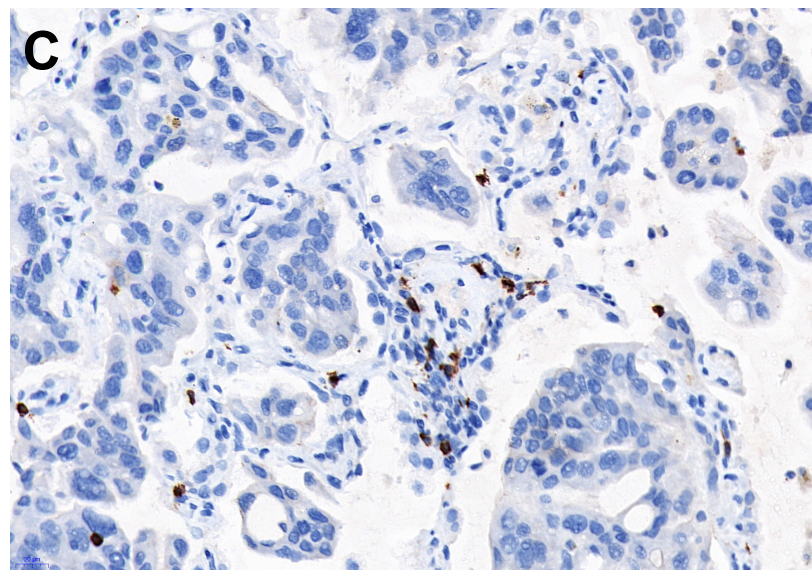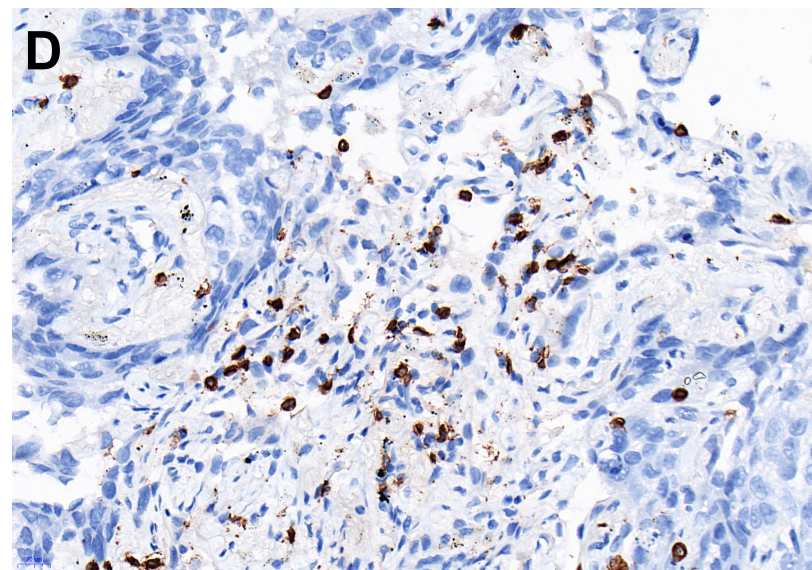

Supplement: Supplementary file 2 — Additional file 2: Figure S2. Immunohistology of CD8+ and CD4+ T cells in P1 (X400). (A) CD8+ T cells of P1A. (B) CD8+ T cells of P1S. (C) CD4+ T cells of P1A. (D) CD4+ T cells of P1S. [file 12920_2020_663_MOESM2_ESM.pdf]
